# Incidence trend for breast cancer among young women in Goiânia, Brazil

**DOI:** 10.1590/S1516-31802010000200007

**Published:** 2010-03-04

**Authors:** Ruffo Freitas, Nilceana Maya Aires Freitas, Maria Paula Curado, Edesio Martins, Carleane Maciel Bandeira Silva, Rosemar Macedo Sousa Rahal, Geraldo Silva Queiroz

**Affiliations:** I MD, PhD. Physician, Gynecology and Breast Service of Hospital Araújo Jorge of the Associação de Combate ao Câncer de Goiás (ACCG); Professor of Gynecology and Obstetrics Department of Federal University of Goiás, Goiânia, Goiás, Brazil.; II MD. Physician, Radiotherapy Service of Hospital Araújo Jorge, Associação de Combate ao Câncer de Goiás (ACCG), Goiás, Brazil.; III MD, PhD. Coordinator of Population-based Cancer Registry of the municipality of Goiânia, Hospital Araújo Jorge, Associação de Combate ao Câncer de Goiás (ACCG), Goiânia, Goiás, Brazil; Head of the Descriptive Epidemiology Production Group, International Agency for Research on Cancer (IARC), Lyon, France.; IV MHSc. Epidemiologist of Population-based Cancer Registry of the municipality of Goiânia, Hospital Araújo Jorge, Associação de Combate ao Câncer de Goiás (ACCG), Goiânia, Goiás, Brazil.; V MD. Professor of Gynecology and Obstetrics, Universidade Federal de Goiás (UFG), Goiânia, Goiás, Brazil.

**Keywords:** Breast neoplasms, Incidence, Epidemiology, Community health planning, Brazil, Neoplasias da mama, Incidência, Epidemiologia, Planejamento em saúde comunitária, Brasil

## Abstract

**CONTEXT AND OBJECTIVE:**

It has been suggested that there has been a large increase in breast cancer incidence among young women over the last decade. The aim of this study was to describe the incidence of breast cancer among young women up to 39 years of age in Goiânia, between 1988 and 2003, and to compare this with other age groups.

**DESIGN AND SETTING:**

Retrospective study using the database of the Population-based Cancer Registry of Goiânia, State of Goiás, Brazil.

**METHODS:**

The incidence was calculated according to age groups: up to 39 years, 40 to 59 years and 60 years and over. Average annual percentage changes (AAPCs) were estimated for the different age groups using Poisson regression.

**RESULTS:**

Over this period, 3,310 new cases were recorded. The standardized incidence was 2.89/100,000 in 1988 and increased to 6.37/100,000 in 2003 (R^2^ = 0.52) for the group aged up to 39 years (p < 0.003). For the group from 40 to 59 years old, the incidence was 14.39/100,000 in 1988 and 41.70/100,000 in 2003 (R^2^ = 0.85; p < 0.001). For the group aged 60 years and over, it was 17.62/100,000 and 28.49/100,000, respectively (R^2^ = 0.67; p < 0.001). The AAPCs were 5.22%, 5.53% and 4.54% for the age groups up to 39, 40 to 59 and 60 years and over, respectively.

**CONCLUSIONS:**

The incidence of breast cancer among young women in Goiânia has been increasing significantly, although this change was similar to the increase in other age groups.

## RESUMO

**CONTEXTO E OBJETIVO:**

Tem sido sugerido que há um grande aumento na incidência do câncer de mama em mulheres jovens na última década. O objetivo deste estudo é descrever a incidência do câncer de mama em mulheres jovens (até 39 anos) em Goiânia, entre 1988 e 2003, comparando com os demais grupos etários.

**DESENHO E LOCAL:**

Estudo retrospectivo utilizando o banco de dados do Registro de Câncer de Base populacional de Goiânia/Goiás, Brasil.

**MÉTODOS:**

As incidências foram calculadas de acordo com os grupos etários: até 39 anos, 40 a 59 anos e, 60 anos acima. A mudança da média percentual anual (MMPA) foi estimada para os diferentes grupos usando a regressão de Poisson.

**RESULTADOS:**

No período, foram registrados 3.310 novos casos. A incidência padronizada foi de 2,89/100.000 em 1988 tendo aumentado para 6,37/100.000 em 2003 (R^2^ = 0,52) para o grupo com até 39 anos (p < 0,003). Para o grupo de 40 a 59 anos a incidência foi de 14,39/100.000 em 1988 e de 41,70/100.000 em 2003 (R^2^ = 0,85; p < 0,001); para o grupo de 60 anos e acima foi de 17,62/100.000 e 28,49/100.000, respectivamente (R^2^ = 0,67; p < 0,001). A MMPA foi de 5,22%, 5,53% e 4,54% para os grupos etários de até 39 anos, 40 a 59 e 60 ou mais, respectivamente.

**CONCLUSÕES:**

A incidência de câncer de mama em mulheres jovens em Goiânia vem aumentando significativamente, entretanto esse aumento é semelhante ao observado nos demais grupos etários.

## INTRODUCTION

Over recent years, breast cancer has been the type of malignant neoplasia of greatest incidence and greatest mortality among Brazilian women.^1-3^ In contrast, despite behavioral changes in Oriental countries, the incidence of breast cancer among postmenopausal women is still lower in those countries than it is in Western countries, since postmenopausal cancer is more related to hormonal exposure and lifestyle-related factors. On the other hand, at the menacme, the incidence in Oriental and Western countries is similar, since there is greater involvement of genetic factors. Such factors contribute primarily to breast cancer among young women.^4^

Also over recent years, the Brazilian media has been reporting that significant increases in breast cancer are occurring among young women of up to 35 years of age.^5,6^ This news has given rise to concern and alarm among the Brazilian public.

## OBJECTIVE

Because of the lack of information on young Brazilian women coming from population-based cancer registries, we conducted the present study with the aims of ascertaining the trend of breast cancer incidence among young women in the city of Goiânia and comparing it with the incidences among other age groups, between the years 1988 and 2003.

## METHODS

After gaining approving for this study from the Research Ethics Committee of Hospital Araújo Jorge, Goiás Anticancer Association (Associação de Combate ao Câncer de Goiás, ACCG), information was sought from the database of the Population-based Cancer Registry (PBCR) of the city of Goiânia. Only women living in Goiânia with a diagnosis of breast cancer confirmed by histological examination were included in this study. These data were obtained by the PBCR through active collection of breast cancer diagnoses from all anatomopathology laboratories in the city, in accordance with a workflow scheme that has been published previously.^3,7^

### Age group stratification and incidence calculation

The patients were divided into age groups according to the following distribution: from 20 to 39 years old (considered to be young women), from 40 to 59 years old and from 60 years old onwards. To calculate the incidence rates for each year, previously established formulas were used.^1,8-10^ The female population of the city of Goiânia that was considered to have been exposed to the risk of cancer for the respective year was defined according to the census populations in the years 1990 and 2000, and according to the extrapolations from these censuses for the other years.^11^

### Statistical analysis

The Statistical Package for the Social Sciences (SPSS) software, version 15, was used for the statistical analysis. Incidence trend curves according to standardized coefficients and year of occurrence were generated through this software. Linear logistic regression analysis was used to obtain correlation coefficients (r) and determination coefficients (r^2^) for adjusting the model of regression equations. The p-trend values were considered significant when P < 0.05.

Poisson’s regression was used, by means of the Jointpoint software (version 3.3.0 of 2008; Statistical Research and Applications Branch, Division of Cancer Control and Population Sciences, National Cancer Institute, United States), to analyze the average annual percentage change (AAPC). Through this, the best model for increases in the coefficient of incidence (standardized for the worldwide population) was established for each age group.

## RESULTS

Between 1988 and 2003, 3,310 new cases of breast cancer in women were registered in the city of Goiânia. From this number, crude annual incidence rates of 22.87/100,000 in 1988 and 68.22/100,000 in 2003 for breast cancer among women were obtained. For comparison, the adjusted incidence rates for the worldwide population of Segi was 36.05 in 1988 and 78.01 in 2003.

By stratifying the sample according to age groups, we could see that there were increases in breast cancer incidence, over the years studied, for all the age groups (**Table 1**). The AAPC was 5.22% for the age group up to 39 years of age, 5.53% for the group from 40 to 59 years of age and 4.54% for the group from 60 years of age onwards (**Table 1**).

**Table 1.** Crude and age-standardized incidence rates for 1988 and 2003, per 100,000 women, in Goiânia

Age group	Crude rate		Age-standardized rate		AAPC (95% CI)
	1988	2003	1988	2003	
20-39	10.31	22.77	2.89	6.37	5.22 (2.91 - 7.54)
40-59	68.54	198.56	14.39	41.70	5.53 (4.50 - 6.62)
60 and over	160.19	259.00	17.62	28.49	4.54 (2.51 - 6.60)
Total	22.87	68.22	36.05	78.01	4.90 (3.84 - 5.92)

AAPC: Average annual percentage change; CI: confidence interval.

**Figure 1.**
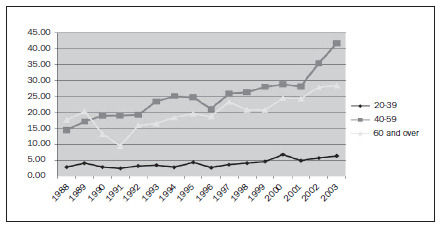
Standardized coefficients of breast cancer incidence in the city of Goiânia, according to the age groups of 20 to 39 years (young women), 40 to 59 years and 60 years and over, for the years 1988 to 2003.

**Figure 2.**
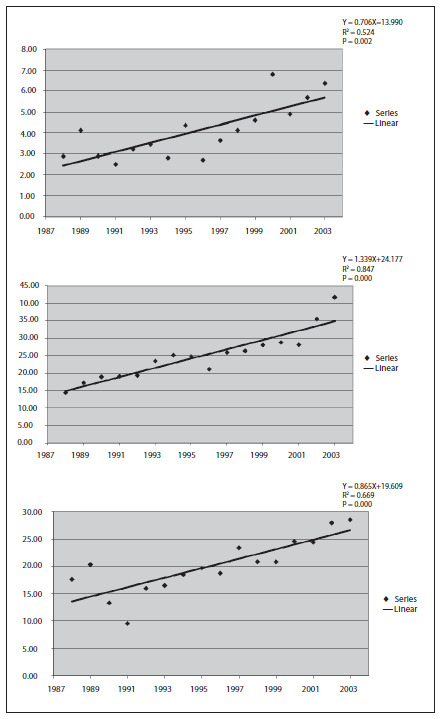
Analysis of standardized incidence trends for breast cancer among women in the city of Goiânia, between 1988 and 2003, according to the age groups of 20 to 39 years (top), 40 to 59 years (middle) and 60 years and over (bottom).

The standardized coefficients of breast cancer incidence, according to the age groups of 20 to 39 years (young women), 40 to 59 years and 60 years onwards, for the years 1988 to 2003, are shown in **Figure 1**. From trend analysis, significant increases in breast cancer incidence rates could be seen in all age groups (**Figure 2**).

## DISCUSSION

Despite large regional variations, the incidence of breast cancer is high throughout the world.^10^ According to data from Globocan 2002, the estimated number of breast cancer cases around the world was 1,152,161, corresponding to a crude incidence rate of 37.4, standardized incidence rate of 37.5 and accumulated risk of 2.6% for women aged 0 to 64 years.^10,12^

In Brazil, 37,528 cases were registered in 2002, corresponding to a crude incidence rate of 42.5, standard incidence rate of 46.0 and accumulated risk of 3.2% for women aged 0 to 64 years.^12^ There may have been some bias in these previously published data, considering that they were furnished by only the five cancer registries that existed at that time, and not the 23 registries that now exist in Brazil.^2^

The data generated from the Population-based Cancer Registry of Goiânia is consistent and have followed the standards described by International Agency for Research on Cancer (IARC)^10^ since 1988 when reliable figures began to be released.^1,3^ We observed in an earlier study that the incidence of breast cancer in the female population of the city of Goiânia had undergone a slight increase, such that the crude annual incidence rate for female breast cancer went up from 21.06/100,000 in 1988 to 41.02/100,000 in 2002 and the standardized incidence went up from 31.88 in 1988 to 51.35 in 2002, with an expected increase of five new cases per 100,000 women per year.^7^ Comparing this incidence with what has been found in other localities, the trend that it followed resembled the findings in some industrialized countries.^4,8,10^ The incidence rates presented in this study differ slightly from the general incidence rates that we published previously,^7^ given that in the present study, we used estimated extrapolations between censuses for the population in each year, whereas in the previous study we used the census population from the Brazilian Institute for Geography and Statistics (Instituto Brasileiro de Geografia e Estatística, IBGE).^11^

In another Brazilian study conducted in Sao Paulo, the presumed prevalence of suspected and highly suspected breast cancer lesions in the population was 0.6%, from studying mammograms from a screened population of 139,945 women.^13^ This figure corroborates the hypothesis that the increase in the incidence of breast cancer, particularly in the age group from 40 to 59 years, may be partially explained by the implementation of population screening programs using mammography. Through these, subclinical lesions that perhaps would not have become clinically detectable, until the patient’s death due to some other cause, can be diagnosed.^4,14-16^

It has been observed that certain risk factors influence the incidence of breast cancer within different age groups.^4,17,18^ For postmenopausal women, increases in body mass index lead to increased risk of breast cancer.^4,15,18^ On the other hand, younger women aged between 15 and 30 years who have not had any child are more predisposed towards breast cancer through exposure to the action of carcinogenic agents such as ionizing radiation.^17,19,20^ This is possibly due to the existence of an initial risk window caused by immaturity of the mammary epithelium, from the time of the menarche to the first pregnancy carried to full term.^21^ Other factors associated with increased incidence of breast cancer that have been well established include: alcoholism, hormone therapy, small numbers of children and even birth of the first child after the age of 30 years.^4,18,22,23^ In addition, there are some other factors that are less clearly established, such as the use of hormonal contraceptives and stress.^8,4,18,23^

The significant increase in breast cancer among young women up to the age of 35 years that has been reported in the media,^5,6^ which has given rise to concern and alarm among the Brazilian public, was extracted from data that originated from a cancer hospital-based registry. Therefore, it was necessary to confirm this information using data from a population-based cancer registry. In the present study, this trend was confirmed. Nevertheless, it needs to be noted that the mean annual increase in the group of young women was similar to the means in the other age groups, thus showing that the results published previously should be viewed with caution. The fact that the increase in incidence for young women was smaller than the increase among those aged 40 years or over, as shown by the AAPC, can be explained by the continual use of opportunistic screening programs that have been carried out yearly in the city of Goiânia since the 1990s.

The results presented here have also been observed in some European countries,^24,25^ in which slight increases in breast cancer incidence among young women, ranging from 2.2% to 3.4% per year, have been shown. In the United States, through data from the Surveillance, Epidemiology and End Results (SEER) program, a slight increase in the incidence of in situ carcinoma has been observed among young women, particularly since the 1980s, with a reduction in invasive carcinomas that has also been slight.^26^

Contrary to the abovementioned studies, growth in the incidence rate of 8.7% per year was observed in Geneva, Switzerland, between 2002 and 2004. This rate was greater than what had previously been observed in that city.^27^ The authors of that study raised the hypothesis that this elevation had occurred mainly through increased use of mammary magnetic resonance imaging.^27^ This technique started to be used in Goiânia in the year 2005, and therefore none of the cases in the present study were detected by means of this imaging method.

This increase is possibly connected not only with aging, but also with changes in habits of life among our population. Another possible explanation would be the improvements in the programs of opportunistic screening that have been implemented in Goiânia, including educational advice for the female population and better access to health services.

The present study has shown that it was possible to establish different patterns of increased incidence, according to the age group. Over the 16-year period studied, it was found that there was a pattern of increasing incidence of breast cancer among women aged 20 to 39 years, but that this increase was similar to the increases seen in the age groups of patients aged 40 years and over. These results indicate that there might not be any particular cause influencing breast cancer incidence among young women in Goiânia.
